# Subphenotyping of prediabetes with a phenotypic tree model and its correlations with outcomes: Insights from two Chinese cohorts

**DOI:** 10.1515/jtim-2026-0034

**Published:** 2026-04-27

**Authors:** Jia Zhang, Jing Ma, Zijian Tian, Yan Zhao, Jian Shao, Guogang Xu, Kaixin Zhou, Qi Pan, Weihao Wang, Lixin Guo

**Affiliations:** Department of Endocrinology, Beijing Hospital, National Center of Gerontology, Institute of Geriatric Medicine, Chinese Academy of Medical Sciences, Beijing, China; Health Management Institute, The Second Medical Center & National Clinical Research Center for Geriatric Diseases, Chinese PLA General Hospital, Beijing, China; Guangzhou Laboratory, Guangzhou International Bio Island, Guangzhou, Guangdong Province, China; Peking University Fifth School of Clinical Medicine, Beijing, China; Graduate School of Peking Union Medical College, Chinese Academy of Medical Sciences, Beijing, China

**Keywords:** prediabetes, subphenotyping, outcomes, progression, type 2 diabetes

## Abstract

**Background and Objectives:**

To investigate whether stratifying participants with prediabetes using the Scottish tree model could identify their risks of developing diabetes and related outcomes.

**Methods:**

The current study enrolled subjects with prediabetes at baseline from the China Health and Retirement Longitudinal Study and the Chinese follow-up cohort, comprising 10,165 and 4,090 participants, respectively. We assigned individuals to the Scottish tree using the mapping function based on nine phenotypic variables, including HbA1c, BMI, blood lipids, liver enzyme, creatinine, and blood pressure. Cox proportional hazards regression models were utilized to assess the relationships between dimensions on the tree and future diabetes, renal and cardiovascular diseases, and death from all causes.

**Results:**

Our findings showed that individuals in the upper-right branch of the tree exhibited the increased risk of progression to subclinical atherosclerosis, cardiovascular disease, microalbuminuria, and all-cause mortality, potentially linked to elevated blood pressure, dyslipidemia, and body fat percentage. Individuals in the right part of the tree were more likely to progress to the onset of type 2 diabetes and chronic kidney disease, which was possibly associated with insulin resistance, triglycerides, and blood pressure.

**Conclusions:**

The phenotypic tree model could be incorporated into future risk stratification and treatment plans to better benefit individuals with prediabetes.

## Introduction

Prediabetes is a metabolic condition marked by elevated blood glucose levels, but has not yet reached the diagnostic threshold for diabetes.^[1]^ Prediabetes is a detrimental cardiometabolic state in relation to elevated risk of both microvascular and macrovascular complications.^[2]^ Substantial research has shown that long-term diabetes complications can manifest in individuals with prediabetes.^[3]^

The most effective strategy for managing prediabetes is intensive lifestyle interventions, which not only help prevent the progression to diabetes but also are associated with a reduction in all-cause mortality during the long-term follow-up.^[4-6]^ However, there are several challenges in managing the progression from prediabetes to diabetes. One challenge derives from the current screening criteria, which rely solely on glucose parameters, lack precision, and have low specificity. As a result, up to 35% of adults in certain populations are classified as having prediabetes, yet only a small proportion (5%–10%) are expected to progress to type 2 diabetes (T2D) annually,^[1]^ leading to substantial increases in the costs associated with prevention efforts.^[7]^ The term “prediabetes” is criticized, as a significant proportion of individuals with prediabetes spontaneously return to normal glucose levels,^[8]^ suggesting that intervention may be unnecessary in these cases. Conversely, despite the implementation of interventions, a considerable proportion of participants in the intervention groups ultimately progressed to diabetes.^[9]^ These issues likely stem from the heterogeneous nature of prediabetes, both in its underlying mechanisms and in its progression to diabetes and related diseases. A deeper understanding of this heterogeneity, along with more precise phenotyping of prediabetes, could enhance the accuracy of risk stratification and management. By classifying prediabetes into biologically comparable groups, physicians could better identify subpopulations that respond differently to lifestyle interventions.

To address the aforementioned issues, Wagner *et al*. and Zheng *et al*. categorized individuals at risk for T2D by combining a variety of phenotypic markers, both glycemic and non-glycemic, to identify subgroups with differing risks of developing T2D and associated complications.^[10,11]^ However, classifying individuals into discrete clusters is suboptimal, as the risk of progression to diabetes is a continuum and the nature of the continuous gradient of this disease must be considered.^[12,13]^ In the area of new-onset T2D, Nair *et al*. developed a phenotypic tree model from the Scottish cohort to visualize and combine glycemic and non-glycemic measures, centered around a molecular taxonomy that stratifies and positions individuals on two-dimensional coordinates according to key pathophysiological underpinnings and metabolic profiles driving the progression of cardiometabolic outcomes.^[14]^ This phenotypic tree model could illustrate how metabolic phenotypes transition smoothly, reflecting the continuous distribution of metabolic traits.

Here, we employed the phenotypic tree, which was developed from the Scottish cohort, for the population with prediabetes to incorporate and visualize phenotypic characteristics in a two-dimensional, tree-like structure. We hypothesized that subphenotyping individuals with prediabetes based on the phenotypic tree is related to the subsequent risk of T2D and related diseases, providing potential strategies for the classification and intervention of prediabetes.

## Methods

### Study population

This multi-cohort study included two cohorts from China, including the Chinese follow-up cohort and the China Health and Retirement Longitudinal Study (CHARLS).

The Chinese follow-up cohort was derived from the Health Management Institute, the Second Medical Center of Chinese PLA General Hospital from 2009 to 2023. This cohort comprised 15,038 individuals with prediabetes who participated in longitudinal physical examination visits, including a detailed medical history, demographic characteristics, anthropometric measurements of body composition, laboratory evaluations of biochemical and metabolic parameters, as well as measurements of brachial-ankle pulse wave velocity (ba-PWV).

The CHARLS, launched in 2011, is an ongoing nationally representative longitudinal cohort, that gathers high-quality data through a structured questionnaire in the Chinese population.^[15]^ In CHARLS 2011, a total of 17,705 participants aged at least 45 years of age were enrolled in the survey and followed up every 2 years after the baseline. This cohort analysis was conducted with the 2011 wave 1 as the baseline, and follow-up data from the 2013 wave 2, 2015 wave 3, and 2018 wave 4. Detailed information on the study design for CHARLS has been provided previously.^[15,16]^

Briefly, in the current study, we recruited population with prediabetes aged 20 years and older in the Chinese follow-up cohort and CHARLS. According to the American Diabetes Association criteria, prediabetes was defined as meeting the following criteria without a diagnosis of diabetes or the use of hypoglycemic medications: fasting blood glucose (FBG) ≥ 5.6 and < 7.0 mmol/L, or HbA1c ≥ 5.7% and < 6.5%.^[17]^

**Figure 1:**
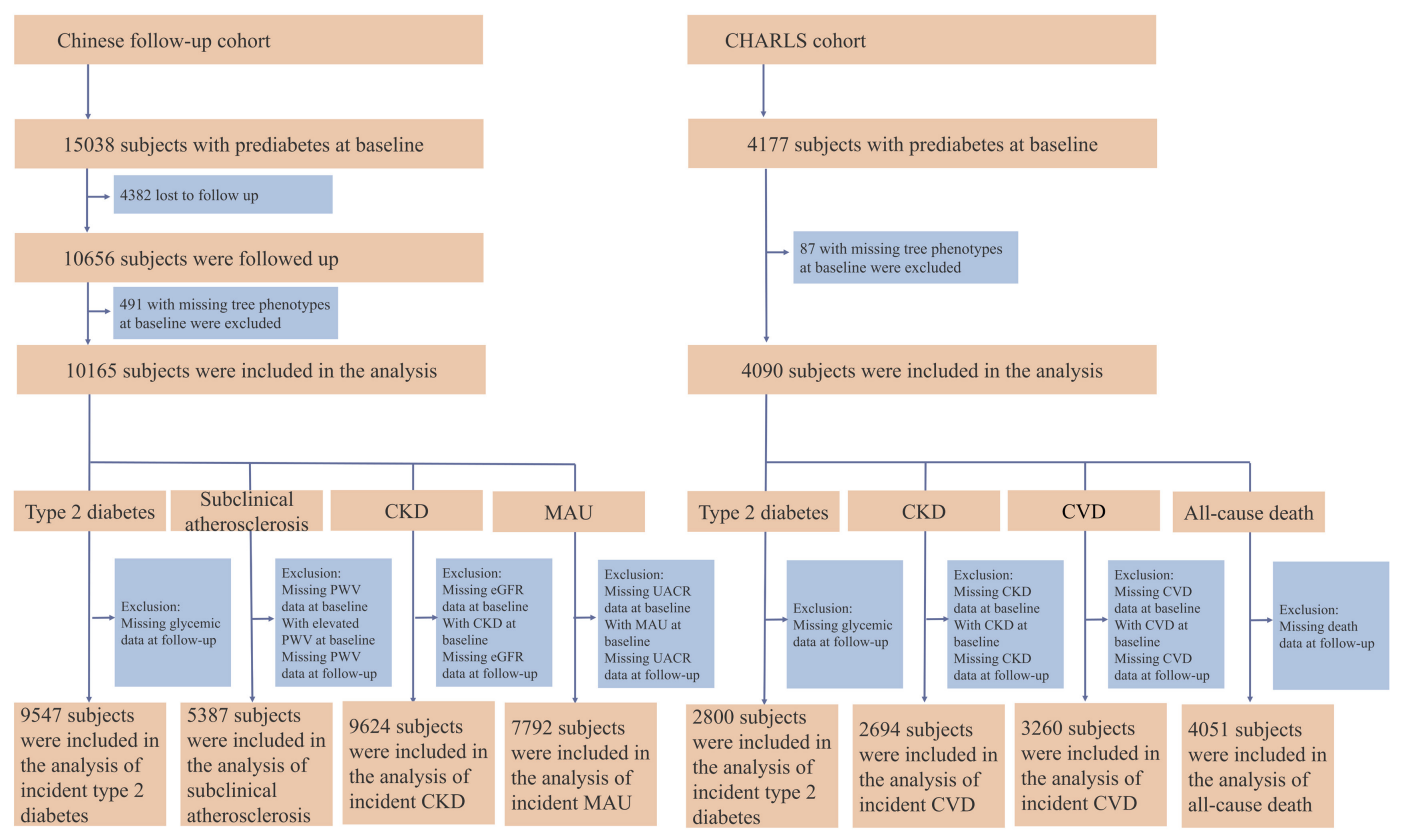
The flowchart of inclusion and exclusion in two Chinese cohorts.

The selection procedure of the study population was displayed in Figure 1. After excluding the individuals without follow-up data or missing tree phenotypes, 10,165 and 4090 subjects from the Chinese follow-up and CHARLS cohorts were enrolled, respectively. Subsequently, participants were excluded based on the following criteria: diagnosis of outcomes at or prior to the baseline survey; missing outcomes information at baseline or follow-up; lost to follow-up.

The Chinese follow-up cohort and CHARLS were approved by the Ethics Review Committees of the Beijing Hospital and Peking University, respectively. The process in this study was approved by the Ethical Committee of Beijing Hospital (2025BJYYEC-KY039-01). The need for written informed consent was waived due to the retrospective design of the study.

### Tree phenotypes and other variables

The nine tree phenotypes including systolic and diastolic blood pressure (SBP and DBP, respectively), high-density lipoprotein cholesterol (HDL-C), triglyceride (TG), total cholesterol (TC), alanine transaminase (ALT), creatinine, body mass index (BMI) and HbA1c were extracted from two cohorts. We substituted the ALT levels in the CHARLS study using the mean value from the Chinese follow-up cohort (25.8 IU/ L), as previous research suggested that the impact of ALT on the positions of the phenotypic tree was minimal.^[14]^ To assess the insulin resistance and β-cell function, we calculated the homoeostasis model assessment of insulin resistance (HOMA-IR) using fasting insulin (μU/mL) × FBG (mmol/L)/22.5, and homoeostasis model assessment β of cell function (HOMA-B) by: (20 × fasting insulin [μU/mL])/(FBG [mmol/L] – 3.5). We derived the waist-to-hip ratio (WHR), body fat percentage, apolipoprotein A (apo A), apo B, and apo E from the Chinese follow-up cohort.

### Outcomes and follow-up

Type 2 diabetes was defined as an HbA1c ≥ 6.5% or a FBG ≥ 126 mg/dL. Subclinical atherosclerosis was determined by a ba-PWV > 1486 cm/s (the highest quantile of the baseline ba-PWV) during follow-up in individuals with a baseline ba-PWV ≤ 1486 cm/s.

To assess the onset of kidney disease, chronic kidney disease (CKD) was determined based on eGFR less than 60 mL/min/1.73 m^2^,^[18]^ and microalbuminuria (MAU) was defined as urinary albumin/creatinine ratio (UACR) ≥ 30 mg/ g. The eGFR was calculated through the Modification of Diet in Renal Disease (MDRD) Study equation.^[19]^

To assess cardiovascular disease (CVD) in the CHARLS, participants were asked if a doctor had ever diagnosed them with heart disease (including myocardial infarction, coronary heart disease, angina, chronic heart failure, or other heart problems) or stroke. Those who answered “yes” were diagnosed with CVD. Incident CVD was defined as those without CVD at wave 1 but developed it by waves 2–4.

All-cause death in CHARLS was ascertained through death certificates or medical records extracted during waves 2–4.^[20]^ For those with an exact time of death (recorded in wave 2), the time-to-event was determined as the interval from wave 1 to the exact date of death. For those without an exact time of death, the time-to-event was calculated as the sum of the interval from baseline to the pre-death interview and the median time between the pre-death interview and the death interview.

The follow-up endpoint was defined as the date of event occurrence or censoring (lost to follow-up or study termination).

### Statistical analyses

Continuous variables were presented as mean [standard deviation (SD)], and categorical variables were presented as number (percentage). Participants in the current analysis were positioned on the phenotypic tree based on HDL-C, TC, TG, HbA1c, BMI, ALT, creatinine, SBP and DBP at baseline through a mapping function.^[14]^ The phenotypic tree model was developed using the DDRTree algorithm, implemented in the Monocle package. DDRTree embeds high-dimensional data into a two-dimensional manifold while preserving global structures in the form of a minimal spanning tree. The phenotypic profiles of individuals, defined by nine clinical variables, were reduced into a two-dimensional representation by the DDRTree algorithm, with each individual assigned to a specific position in this reduced space. The principal trunk represents a central cluster with mixed phenotypes, whereas distal branches capture more homogeneous and distinct phenotypic profiles. Dimension 1 and Dimension 2, derived from nine clinical profiles through dimension reduction, represent each participant’s coordinates on the two-dimensional phenotypic tree.

The evaluation of continuous outcomes and their corresponding predicted values on the phenotypic tree were conducted using linear regression models. For binary outcomes, their predicted odds ratios (ORs) were also mapped to the tree, with ORs calculated from logistic regression models. Time-to-event outcomes, along with their predicted hazard ratios (HRs), were analyzed across the tree using Cox regression models. The gradients of ORs and HRs were both referenced to one representative 50-year-old male at coordinates (0, 0). All models were adjusted for age and gender.

We further mapped the glycemic status (FBG ≥ 5.6 mmol/L only; HbA1c ≥ 5.7% only; FBG ≥ 5.6 mmol/L and HbA1c ≥ 5.7%), to the reference tree and fitted multinomial regression models to examine the distributions of glycemic categories on the tree, using FBG ≥ 5.6 mmol/ L as the reference group.

R (version 4.2.0) was used for all analyses. A two-sided *P*-value < 0.05 was considered statistically significant.

## Results

### Clinical characteristics of study population

A total of 4,090 participants with prediabetes > 20 years of age from the CHARLS study, and 10,165 individuals from the Chinese cohort were enrolled in the current study. The median follow-up time was 9 years for death, 7 years for CVD, 4 years for T2D and CKD in CHARLS, as well as 3.05 for T2D, 3 years for atherosclerosis progression, 3.1 years for MAU, and 3.2 years for CKD in the Chinese follow-up cohort.

The overall clinical features at baseline were displayed in Table 1 and Supplementary Table S1. The proportion of participants taking antihypertensive medications and blood pressure control in the two cohorts were described in Supplementary Table S2. Of note, participants who received antihypertensive medications had suboptimal blood pressure control.

**Table 1:** Characteristics of study population from The Chinese follow-up cohort and CHARLS

Characteristics	The Chinese follow-up cohort	CHARLS cohort
*N*	10,165	4090
Age (years)	50.18 ± 8.38	59.46 ± 9.46
Male (%)	7215 (70.98)	1872 (45.77)
SBP (mmHg)	122.59 ± 17.03	130.4 ± 21.36
DBP (mmHg)	80.24 ± 10.84	75.72 ± 12.07
HDL-C (mmol/L)	1.22 ± 0.32	1.33 ± 0.4
Total (mmol/cholesterol L)	4.92 ± 0.92	5.12 ± 1.01
Triglycerides (mmol/L)	1.88 ± 1.19	1.47 ± 0.95
HbA1c (%)	5.76 ± 0.3	5.19 ± 0.42
BMI (kg/m^2^)	25.73 ± 3.06	23.67 ± 3.83
ALT (IU/L)	25.78 ± 15.82	——
Creatinine (mmol/L)	70.19 ± 13.79	69.36 ± 22.95
HOMA-B	2.93 ± 1.65	——
HOMA-IR	115.2 ± 60.86	——

Continuous variables were presented as mean ± SD, and categorical variables were presented as *N* (%). CHARLS: China Health and Retirement Longitudinal Study; SBP: systolic blood pressure; DBP: diastolic blood pressure; HDL-C: High-density lipoprotein; BMI: body mass index; ALT: alanine transaminase creatinine; HOMA-B: homoeostasis model assessment β of cell function; HOMA-IR: homoeostasis model assessment of insulin resistance.

### Distributions of phenotypes across the tree

The visualization of the gradient for phenotypic characteristics across the tree was provided (Supplementary Figure S1-S3). These two cohorts exhibited a very similar pattern in the visualization charts of tree variables. A substantial proportion of participants were located in the left part of the phenotypic tree, rather than the right part, which may be due to the lower HbA1c levels in prediabetes compared with diabetes, leading to an overall distribution predisposed towards the left. We further investigated the distribution of phenotypic components on the tree by dividing each phenotype into quantiles, revealing that participants in the upper-right branch had the highest levels of SBP, DBP, and TC, while those in the upper-left branch exhibited the highest HDL-C. Participants in the lower-right position were more likely to have elevated BMI and TG levels.

### Correlations between positions across the tree and major outcomes

To explore the phenotypic tree associated with diabetes and other clinical outcomes, we derived CVD, CKD, and all-cause mortality from the CHARLS cohort and atherosclerosis progression, MAU, metabolic indices, and clinical biomarkers from the Chinese follow-up cohort. During the follow-up period, 1280, 1284, 95, and 301 participants with prediabetes developed T2D, subclinical atherosclerosis, CKD, and MAU, respectively. In the analysis for CHARLS, 414, 122, 497, and 485 individuals with prediabetes progressed to T2D, CKD, CVD, and all-cause death, respectively (Table 2).

**Table 2:** The relationships of two dimensions on the tree with type 2 diabetes and its complications

Clinical outcomes	Events/*N*	Dimension 1		Dimension 2	
		HR/OR (95% CI)	*P* value	HR/OR (95% CI)	*P* value
Chinese follow-up cohort					
Type 2 diabetes	1280/9547	2.44 (2.17, 2.75)	<0.001	0.95 (0.83, 1.08)	0.431
Subclinical atherosclerosis	1284/5387	1.88 (1.62, 2.19)	<0.001	1.75 (1.50, 2.03)	<0.001
CKD	95/9624	1.98 (1.31, 3.01)	0.001	1.03 (0.63, 1.68)	0.918
MAU	301/7792	3.04 (2.43, 3.81)	<0.001	1.43 (1.07, 1.87)	0.014
CHARLS cohort					
Type 2 diabetes	414/2800	1.76 (1.41, 2.18)	<0.001	0.77 (0.59, 0.99)	0.038
CKD	122/2694	1.31 (0.87, 1.91)	0.185	0.81 (0.52, 1.26)	0.338
CVD	497/3260	1.60 (1.35, 1.91)	<0.001	1.32 (1.06, 1.64)	0.014
All-cause mortality	485/4051	1.38 (1.16, 1.65)	<0.001	1.57 (1.26, 1.97)	<0.001

All models were adjusted for age and gender. CKD: chronic kidney disease; MAU: microalbuminuria; CKD: chronic kidney disease; CVD: cardiovascular disease.

**Figure 2:**
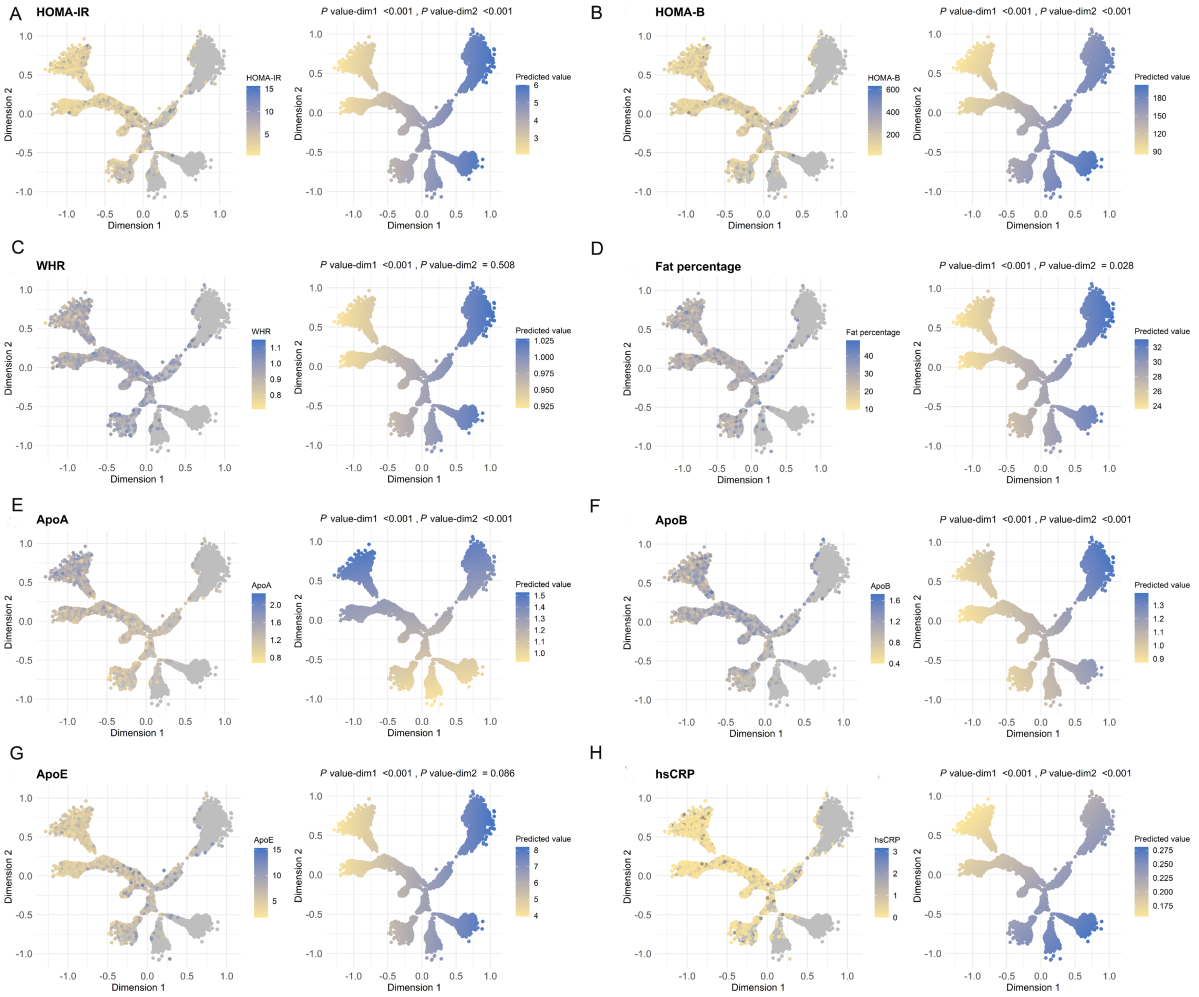
Visualizing the heterogeneity in the metabolic profiles using the phenotypic tree model in the Chinese follow-up cohort. Left panel: each dot represents the position of one participant. Right panel: predicted value for metabolic profiles at baseline. (A) HOMA-IR: homoeostasis model assessment of insulin resistance. (B) HOMA-B: homoeostasis model assessment β of cell function. (C) WHR: waist-to-hip ratio. (D) Body fat percentage. (E) apo A: apolipoprotein A. (F) apo B: apolipoprotein B. (G) apo E: apolipoprotein E. (H) hsCRP: high-sensitivity C-reactive protein.

Insulin resistance and β-cell function, as assessed by HOMA-IR and HOMA-B, showed a similar pattern, being positively linked to the x- and y-axes, with the highest predicted value in the lower-right part of the tree and the lowest in the upper-left region (Figure 2A and 2B). The WHR was only correlated with dimension 1 (Figure 2C). Although BMI was most prominent in the lower-right section of the tree, the body fat percentage (Figure 2D) predominantly clustered in the upper-right branch. This may be attributed to the worse ability of BMI to precisely capture fat distribution.^[21]^ Apo B and apo A could function as indicators for the total count of atherogenic and antiatherogenic particles, respectively. Previous research suggested that these markers offered superior prognostic value for CVD compared to conventional lipid panels.^[22,23]^ The apo A value showed a negative correlation with the x-axis and a positive association with the y-axis (Figure 2E). Conversely, apo B was positively correlated with both axes (Figure 2F), while apo E was notably higher in the right-hand section of the tree (Figure 2G). The inflammatory marker, high-sensitivity C-reactive protein (hs-CRP), was primarily concentrated in the lower-right region (Figure 2H).

**Figure 3:**
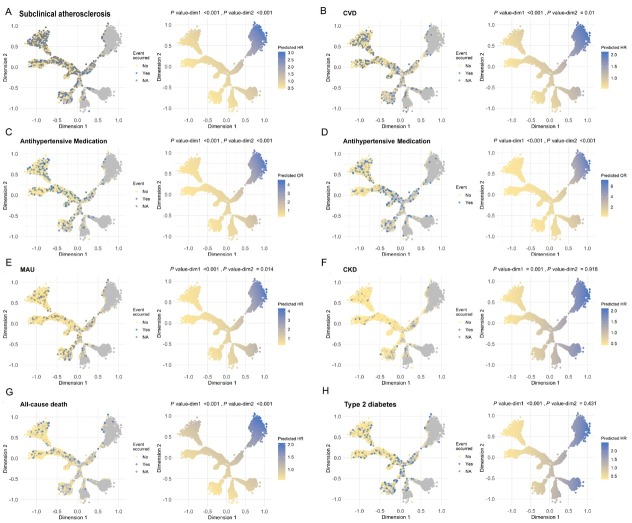
Identifying the heterogeneity in the progression to diabetes and outcomes using the phenotypic tree model in the Chinese follow-up cohort and CHARLS. Left panel: each dot represents the position of one participant. Right panel: predicted HR for time-to-event outcomes and OR for binary outcomes. (A) subclinical atherosclerosis in the Chinese follow-up cohort. (B) CVD in CHARLS. (C) antihypertensive medication in the Chinese follow-up cohort. (D) antihypertensive medication in CHARLS. (E) MAU in the Chinese follow-up cohort. (F) CKD in the Chinese follow-up cohort. (G) all-cause death in CHARLS. (H) type 2 diabetes in the Chinese follow-up cohort. CVD: cardiovascular disease. MAU: microalbuminuria. CKD: chronic kidney disease; CHARLS: China Health and Retirement Longitudinal Study.

We further assigned individuals with prediabetes to the treelike structure and conducted analyses on the relationships of different positions across the tree with the risk of progressing to diabetes, and cardiovascular and renal diseases, as well as all-cause death (Supplementary Figure S4). The risk of developing subclinical atherosclerosis in the Chinese follow-up cohort, a precursor of CVD, was positively associated with both dimension 1 and dimension 2 (Figure 3A), corresponding to the distributions of SBP, DBP, and body fat percentage. As for CVD in CHALRS, individuals who located in the upper right branch exhibited the highest risk of incident CVD (Figure 3B), showing an elevated risk in subpopulations at the upper-right branch. Interestingly, individuals in the upper-right region had the highest proportion receiving antihypertensive medication than those in other regions, both in the Chinese follow-up cohort (Figure 3C) and in CHARLS (Figure 3D). Nevertheless, those individuals on antihypertensive medication still showed suboptimal blood pressure control compared to others (Supplementary Table S2), which aligned with the patterns of worse cardiovascular outcomes. With respect to renal disease, participants located in the upper-right region were more likely to develop MAU, defined as a UACR greater than 30 (Figure 3E), resembling the distribution observed for subclinical atherosclerosis and clinical CVD, and linking to increased SBP and DBP. The highest risk for new-onset CKD (Figure 3F), indicated by an eGFR of less than 60 mL/min/1.73 m^2^, was observed in the right region, mirroring the distribution of both higher blood pressure and insulin resistance. The risk of all-cause mortality was maximum in the upper-right, mirroring the distribution patterns of cardiovascular and renal events (Figure 3G). The participants allocated to the entire right part of the tree were prone to develop type 2 diabetes during the follow-up (Figure 3H). In the Chinese follow-up cohort, participants located in the lower-left region were more likely to be using lipid-lowering medications at baseline, whereas in the CHARLS cohort, such individuals were predominantly distributed in the lower-right region (Supplementary Figure S5 and S6).

**Figure 4:**
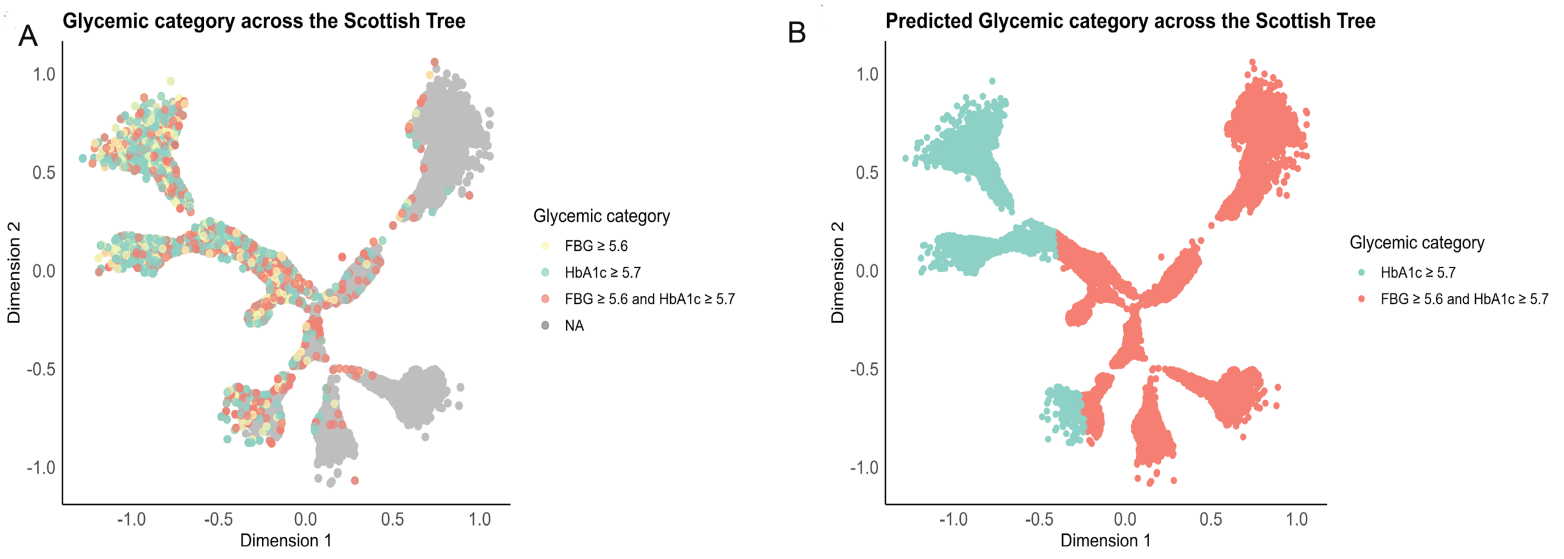
Distribution of glycemic status across phenotypic tree model. (A) The distributions of glycemic category on the phenotypic tree (B) Predicted glycemic category on the phenotypic tree. FBG: fasting blood glucose; HbA1c: hemoglobin A1c.

Moreover, the glycemic categories were presented in Figure 4, revealing that the identification of the population with prediabetes who were allocated to the right part of the tree was sensitive to a combination of FBG and HbA1c.

## Discussion

In the current analysis of population with prediabetes from two Chinese cohorts, we employed the reference tree and mapping function developed from the Scottish cohort to visualize continuous phenotypic heterogeneity of the population with prediabetes, encapsulating intricate and concurrent pathophysiological processes and metabolic multimorbidity. To the best of our knowledge, this study is the first research to visualize and classify the Chinese population with prediabetes using a tree-based approach, reducing nine simple clinical variables into two dimensions, residualized for age and gender, for identifying patients at elevated risk of diabetes and other clinical outcomes, as well as all-cause mortality. Future risks of diseases varied across distributions on the tree. The upper-right branch of the tree had elevated risks of subclinical atherosclerosis, CVD, MAU, and death from all causes, in accordance with the increased levels of SBP, DBP, and TC. Overall, subpopulations across the entire right region of the tree tended to transition into diabetes, corresponding to the high levels of insulin resistance, whereas those in the upper-right part faced elevated risks of progressing to cardiorenal diseases and mortality, possibly in relation to uncontrolled blood pressure and dyslipidemia.

Previous studies conducted in European and Chinese by Wagner and Zheng, respectively, employed data-driven cluster analysis using phenotypic variables to categorize patients with prediabetes into six subgroups, each exhibiting different metabolic characteristics and associated risk of disease.^[10,11]^ Their studies offered significant contributions to understanding the diversity within prediabetes. However, their clustering methods categorized individuals based on the resemblance of selected variables, while metabolic factors exhibited substantial variability within individuals. As the number of variables and clusters increased, the stability of cluster assignments often decreased. This phenomenon may be because the changes in cardiovascular metabolic risk factors during follow-up could influence the reclassification of prediabetes, suggesting that categorizing the population into distinct groups may not be appropriate. Similar to their studies, our tree-based method also incorporated multiple phenotypic features. Importantly, our model further displayed smooth transitions of subphenotypes within individuals across the tree, rather than dividing them into distinct categories. Using clinical measures from multiple follow-up visits and observing the transitions in individuals’ positions on the tree, physicians were able to capture individuals’ changes in the risk of diabetes and other clinical outcomes.

Our findings herein showed that individuals predominantly clustered in the upper-right branch of the tree had the highest risk of progression to subclinical atherosclerosis, cardiovascular disease, and all-cause death, which may be related to elevated blood pressure, cholesterol levels, and body fat percentage. Although the subpopulation in the top-right branch exhibited the highest proportion of individuals receiving antihypertensive medication in both the Chinese follow-up and CHARLS cohorts, their blood pressure control remains suboptimal. A pivotal study by Bi *et al*. in China demonstrated that reducing blood pressure to 120 mmHg could significantly decrease the incidence of cardiovascular events among individuals with diabetes.^[24]^ Another study by Liu *et al*. also reported that targeting SBP below 120 mmHg reduced the risk of vascular outcomes, independent of diabetic status, within the Chinese participants.^[25]^ Not only do these two landmark studies, but also our findings, underscore the necessity for more intensive blood pressure lowering in the management and prevention of cardiovascular events, particularly in the Chinese population at high risk for T2D.

It was worth noting that individuals with a higher risk of insulin resistance were positioned in the bottom-right branch, a location that was not in line with the greatest risk of developing outcomes. Previous cluster-based analyses have demonstrated that low insulin secretion, coupled with only moderate insulin resistance, is the predominant characteristic of diabetes subtypes in East Asian populations.^[26,27]^ Genetic evidence further supports the view that β-cell function plays a more central role, compared to insulin resistance, in the onset of diabetes, particularly among the Chinese population.^[28]^ These findings may help explain why individuals at the highest risk for cardiovascular events were positioned in the top-right region of the analysis, which was in relation to uncontrolled blood pressure, rather than in the lower-right region that reflected insulin resistance. The insulin resistance is less pronounced in the progression to diabetes among the Chinese population.^[26,27]^

There have been numerous trials that have demonstrated that targeted interventions for prediabetes can significantly slow the progression to T2D.^[4,5,29]^ However, the implementation of diabetes prevention strategies in clinical settings remains challenging. These interventions are often resource-intensive, face resistance, and are often rejected, particularly when patients with prediabetes are unaware of their elevated blood glucose or when the benefits are not immediately apparent.^[7]^ Moreover, the identification and classification of prediabetes may be overlooked or imprecise, especially when diagnosis is based exclusively on glycemic thresholds, without accounting for other metabolic factors. In addition, prediabetes is a highly heterogeneous metabolic condition, both in terms of its pathophysiology and disease prediction. A deeper understanding of its pathophysiological mechanisms is crucial for more effective risk classification. We hypothesize that these objectives can be achieved through the application of metabolic phenotyping strategies and precise body measurements. With this perspective, our study could visualize the heterogeneity of prediabetes using a two-dimensional tree and offer insights into the differences in the progression to diabetes and other clinical outcomes. The tree model could serve as a new tool for phenotyping prediabetes, and this approach could be translated into personalized risk prediction and prevention strategies for Chinese patients.

## Limitation

Our study had several limitations. First, the diagnosis of prediabetes was based only on FBG and HbA1c levels, without including the 2-hour post-glucose measurement, which introduced bias when illustrating the results. Second, due to the lower glycemic measures of prediabetes versus diabetes, we observed a leftward skew in the distribution of individuals across the tree. However, given the commonalities of metabolic diseases, our tree-based model remains applicable to prediabetes for the purpose of assessing the risk of developing diabetes and stratifying disease. Third, our tree model failed to account for genetic factors or treatment interventions, which possibly limits its utility in complex clinical settings, particularly for individuals who have received interventions or have varying genetic backgrounds. While including routine clinical variables can improve the study’s generalizability, researches still need to investigate novel biomarkers, such as inflammatory cytokines and genetic factors, to better elucidate metabolic heterogeneity and its associations with various diseases. Lastly, the relatively short follow-up duration in both cohorts may limit the ability to capture long-term outcomes. Future studies with extended follow-up are warranted to validate and extend our findings.

## Conclusion

Overall, our study revealed significant metabolic heterogeneity among individuals with prediabetes. The identified subphenotypes on different branches of the phenotypic tree exhibited distinct metabolic profiles and were linked to varying risks of diabetes, cardiorenal disease, and death. With further refinement and validation, the tree model could guide targeted early interventions in individuals with prediabetes most at risk.

## Supplementary Material

Supplementary Material Details
