# Supplementary material for: Subphenotyping of prediabetes with a phenotypic tree model and its correlations with outcomes: Insights from two Chinese cohorts
Source: J Transl Int Med. 2026 Apr 27;14(3):413–22. doi: 10.1515/jtim-2026-0034 (PMC13320526; doi:10.1515/jtim-2026-0034)
Supplement: Supplementary file 1 — Supplementary Material Details [file jtim-2026-0034_sm.pdf]

Supplementary materials

Supplementary Table S1: The associations between two dimensions and clinical parameters in the Chinese follow-up cohort.

	N	**Dimension 1**		**Dimension 2**	
		**Estimate (95% CI)**	***P* value**	**Estimate (95% CI)**	***P* value**
ApoA	10103	-0.041 (-0.05, -0.03)	<0.001	0.28 (0.26, 0.29)	<0.001
ApoB	10102	0.20 (0.19, 0.21)	<0.001	0.09 (0.08, 0.10)	<0.001
ApoE	10086	1.81 (1.74, 1.89)	<0.001	-0.07 (-0.15, 0.01)	0.086
hsCRP	9187	0.04 (0.02, 0.05)	<0.001	-0.04 (-0.05, -0.02)	<0.001
HOMA-IR	9524	1.62 (1.54, 1.69)	<0.001	-0.17 (-0.25, -0.09)	<0.001
HOMA-B	9524	40.9 (37.9, 43.9)	<0.001	-18.2 (-21.3, -15.0)	<0.001
WHR	10131	0.05 (0.045, 0.05)	<0.001	-0.00(-0.00, 0.00)	0.508
Fat percentage	10097	4.01(3.76, 4.26)	<0.001	0.29 (0.03, 0.54)	0.028

All models were adjusted for age and sex. apo A: apolipoprotein A; apo B: apolipoprotein B; apo E: apolipoprotein E; hsCRP: high-sensitivity C-reactive protein; HOMA-IR: homoeostasis model assessment of insulin resistance; HOMA-B: homoeostasis model assessment β of cell function; WHR: waist-to-hip ratio.

Supplementary Table S2: The antihypertensive medication and blood pressure control in two cohorts

**Characteristics**	**The Chinese follow-up cohort**	**CHARLS**
Antihypertensive medication	2051 (21.18%)	808 (19.76%)
SBP (with antihypertensive medication)	131.05 ± 16.21	143.80 ± 22.21
DBP (with antihypertensive medication)	84.77 ± 10.49	81.02 ± 12.43
SBP (without antihypertensive medication)	120.37 ± 16.60	127.09 ± 19.80
DBP (without antihypertensive medication)	79.14 ± 10.66	74.41 ± 11.62
SBP (on the upper-right brunch)	153.84 ± 11.37	170.77 ± 17.00
DBP (on the upper-right brunch)	99.86 ± 7.78	96.15 ± 11.64

SBP: systolic blood pressure; DBP: diastolic blood pressure.

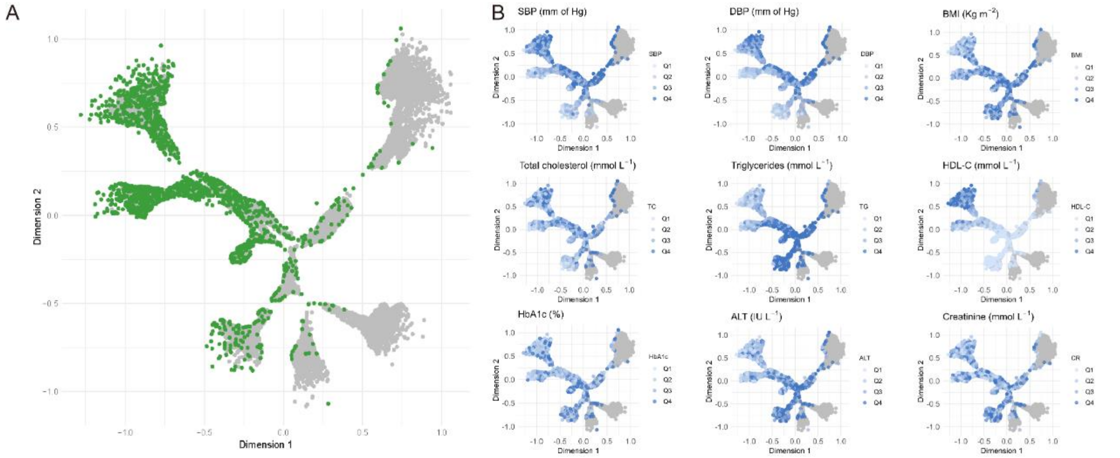

Supplementary Figure S1: The allocation of participants in the Chinese follow-up cohort at baseline. The positions of participants of the Chinese follow-up cohort. (B) The distributions of nine phenotypes across the phenotypic tree.

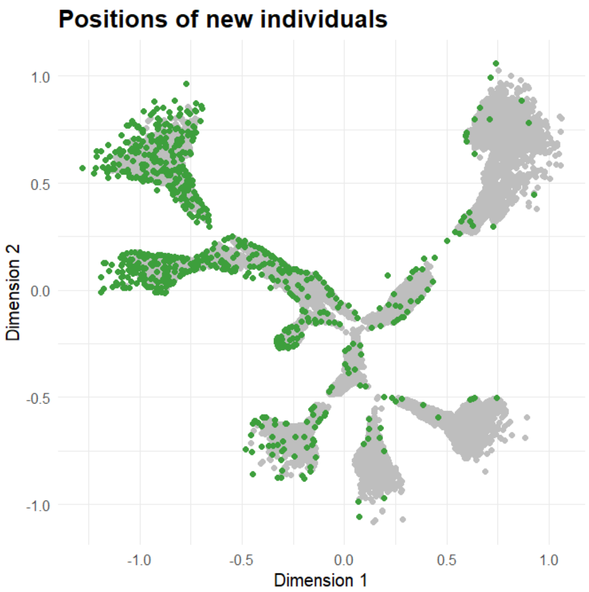

Supplementary Figure S2: The positions of participants of CHARLS at baseline.

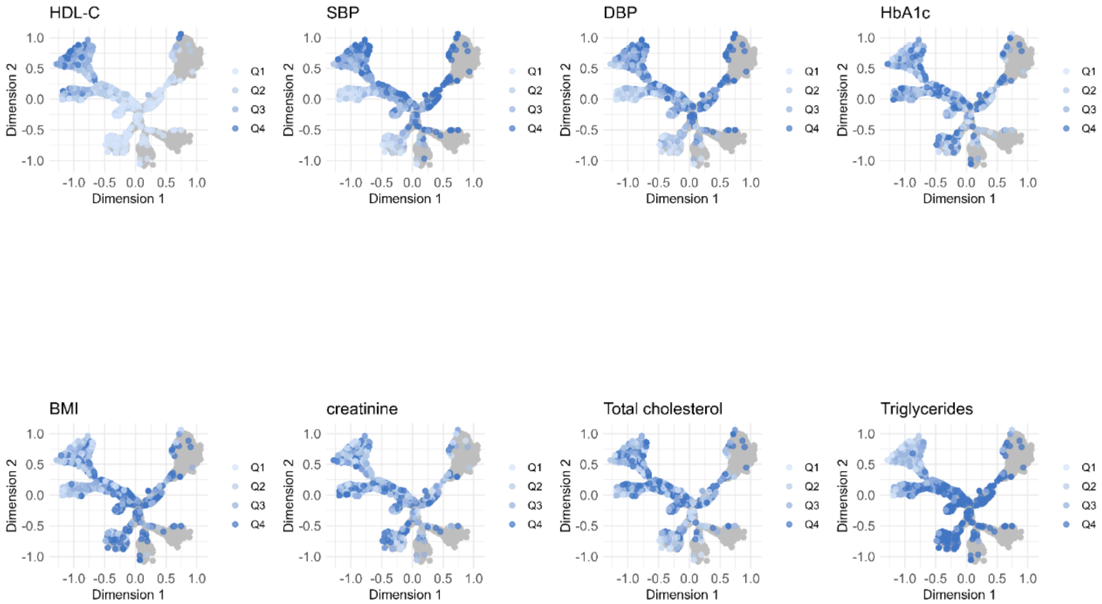

Supplementary Figure S3: The distributions of nine phenotypes for participants in CHARLS at baseline.

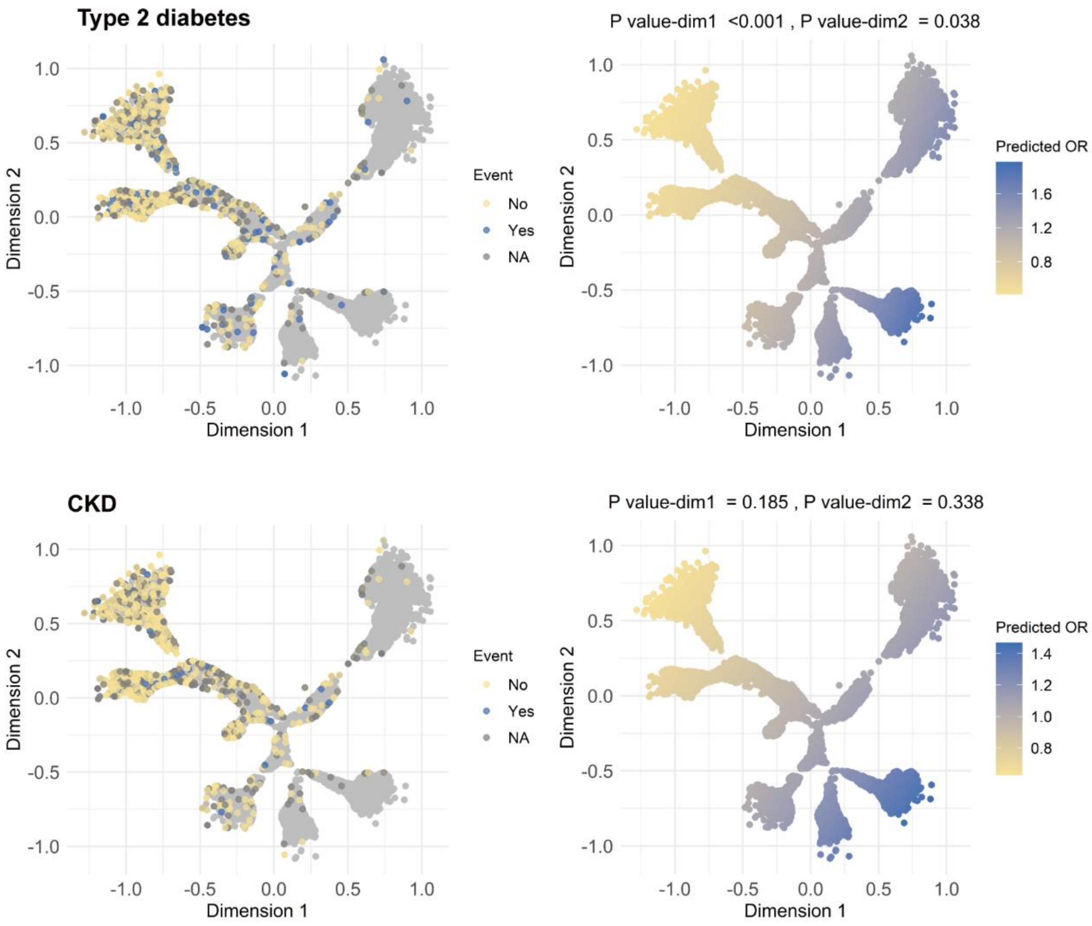

Supplementary Figure S4: The risk of developing type 2 diabetes and chronic kidney disease in CHARLS.

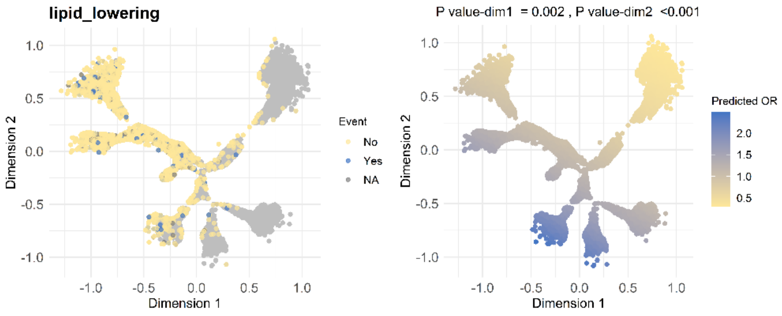

Supplementary Figure S5: Baseline lipid-lowering medication in Chinese follow-up cohort.

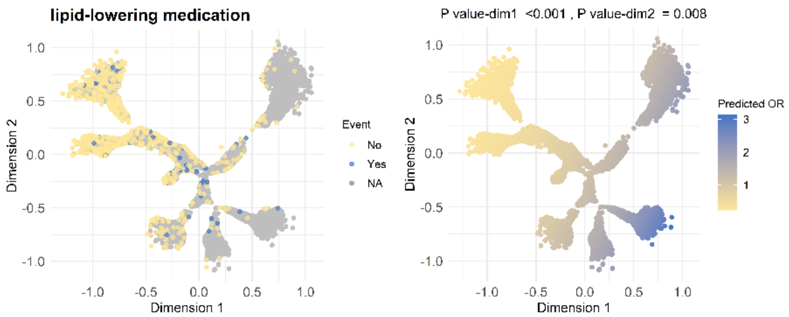

Supplementary Figure S6: Baseline lipid-lowering medication in CHARLS cohort.
